# Editorial: Recent advances in boron neutron capture therapy in radiation oncology

**DOI:** 10.3389/fonc.2024.1398781

**Published:** 2024-05-17

**Authors:** Ling-Wei Wang

**Affiliations:** ^1^Department of Heavy Particles and Radiation Oncology, Taipei Veterans General Hospital, Taipei, Taiwan; ^2^School of Medicine, National Yang Ming Chiao Tung University, Hsinchu, Taiwan

**Keywords:** BNCT (boron neutron capture therapy), malignant melanoma, tumor control probability (TCP), MDSC (myeloid-derived suppressor cells), out-of-field leakage

In this Research Topic, “*Recent Advances in Boron Neutron Capture Therapy in Radiation Oncology*”, there were four papers published. Hsu et al. discussed developing a tumor control probability (TCP) model from a clinical trial done in Taiwan for recurrent head and neck (H&N) cancer. Their analysis showed the generalized Equivalent Uniform Dose (gEUD)–based TCP model (derived from the universal survival curve (USC) model) correlated well with the tumor response and dose distribution of recurrent H&N cancer. The recommended minimal tumor dose (18 Gy-w in a single fraction) can be used as a reference dose for future curative BNCT in this challenging scenario.

The impact of BNCT on the immune system is seldom explored. The second paper by Chang et al. is a basic study investigating the association of M-MDSCs(monocytic myeloid-derived suppressive cells) depletion with the therapeutic effect of BNCT in mice bearing oral squamous cell carcinoma. Myeloid-derived suppressor cells are a heterogeneous population of myeloid cells that appear in the tumor microenvironment (TME) and can modulate immune response. They found that M-MDSCs with CSF-1 receptors were recruited into murine tumors after BNCT, and their number was also increased in peripheral blood. The administration of PLX-3397, a CSF-1 receptor inhibitor, could hinder BNCT-caused M-MDSCs infiltration, prolong mice survival, and activate tumor immunity. This may be useful for the design of future trials combining immunotherapy and BNCT for recurrent H&N cancer.

Accelerator-based BNCT (AB-BNCT) systems installed in hospitals is becoming a reality. There were two papers on this topic corelated with AB-BNCT. The third paper by Igaki et al. showed the result of an acral cutaneous malignant melanoma treated with AB-BNCT at the National Cancer Center Hospital, Tokyo, Japan. The maximum dose delivered to the skin was 18 Gy-Eq. The skin lesion disappeared completely within 12 months. Adverse effects including dermatitis, dry skin, skin hyperpigmentation, and edema were mild (only grade 1). This case report showed that AB-BNCT may be a promising treatment modality for cutaneous malignant melanoma.

The fourth paper by You et al. explored out-of-field leakage from the neutron beam of the AB-BNCT system installed in a hospital in Zhubei, Taiwan. The out-of-field leakage dose, also called a non-target radiation dose, is of concern in AB-BNCT because it causes unnecessary risk of harm to patients. Therefore, it is important to consider out-of-field leakage in beam shaping assembly (BSA) design. Calculation in this study found that the whole-body dose in their system was low. In the near future, we expect to see more studies reporting results from both basic and clinical research on AB-BNCT.

## Author contributions

L-WW: Writing – original draft, Writing – review & editing.

